# American Medicinal Plants

**Published:** 1887-02

**Authors:** 


American Medicinal Plants. By C. F. Millspaugh, M. D. Bœricke & Tafel: New York and Philadelphia.

The fifth fascicle of this exquisitely beautiful work was issued in December last. Among the many plants illustrated our eye was particularly arrested with the following familiar remedies of our materia medica:

Ranunculus Scel., Yellow Mustard, St. John's Wort, Æsculus Glabra, Rag Weed, Lactuca, Collinsonia, Lupulus, and Lachnantes.

The representation of the leaf of Æsculus Glabra is particularly fine.

Why does not *every* homœopathic physician possess himself of this beautiful work? We hope to hear of *all* the subscribers to this journal procuring it.

